# Biofunctional Hyaluronic Acid/κ-Carrageenan Injectable Hydrogels for Improved Drug Delivery and Wound Healing

**DOI:** 10.3390/polym14030376

**Published:** 2022-01-18

**Authors:** Uzma Ijaz, Muhammad Sohail, Muhammad Usman Minhas, Shahzeb Khan, Zahid Hussain, Mohsin Kazi, Syed Ahmed Shah, Arshad Mahmood, Mohammed Maniruzzaman

**Affiliations:** 1 Department of Pharmacy, Abbottabad Campus, COMSATS University Islamabad, Abbottabad 22010, Pakistan; uzmaijaz92@gmail.com (U.I.); syedahmed.shah@superior.edu.pk (S.A.S.); 2 College of Pharmacy, University of Sargodha, Sargodha 40100, Pakistan; usman.minhas@uos.edu.pk; 3 Department of Pharmacy, University of Malakand, Chakdara 18800, Pakistan; shahzebkhan@uom.edu.pk; 4 Discipline of Pharmaceutical Sciences, School of Health Sciences, University of KwaZulu-Natal, Durban 4041, South Africa; 5 Department of Pharmaceutics & Pharmaceutical Technology, College of Pharmacy, University of Sharjah, Sharjah P.O. Box 27272, United Arab Emirates; zhussain@sharjah.ac.ae; 6 Research Institute for Medical and Health Sciences (SIMHR), University of Sharjah, Sharjah P.O. Box 27272, United Arab Emirates; 7 Department of Pharmaceutics, College of Pharmacy, King Saud University, P.O. Box 2457, Riyadh 11451, Saudi Arabia; mkazi@ksu.edu.sa; 8 Department of Pharmaceutical Sciences, The Superior University, Lahore 54600, Pakistan; 9 College of Pharmacy, Al Ain University, Abu Dhabi P.O. Box 112612, United Arab Emirates; arshad.mahmood@aau.ac.ae; 10 Division of Molecular Pharmaceutics and Drug Delivery, Department of Molecular Pharmaceutics and Drug Delivery, College of Pharmacy, The University of Texas at Austin, Austin, TX 78712, USA; m.maniruzzaman@austin.utexas.edu

**Keywords:** bioactive polymers, thermosensitive hydrogel, biomaterials, wound repair and regeneration

## Abstract

The in situ injectable hydrogel system offers a widespread range of biomedical applications in prompt chronic wound treatment and management, as it provides self-healing, maintains a moist wound microenvironment, and offers good antibacterial properties. This study aimed to develop and evaluate biopolymer-based thermoreversible injectable hydrogels for effective wound-healing applications and the controlled drug delivery of meropenem. The injectable hydrogel was developed using the solvent casting method and evaluated for structural changes using proton nuclear magnetic resonance, Fourier transforms infrared spectroscopy, thermogravimetric analysis, and scanning electron microscopy. The results indicated the self-assembly of hyaluronic acid and kappa-carrageenan and the thermal stability of the fabricated injectable hydrogel with tunable gelation properties. The viscosity assessment indicated the in-situ gelling ability and injectability of the hydrogels at various temperatures. The fabricated hydrogel was loaded with meropenem, and the drug release from the hydrogel in phosphate buffer saline (PBS) with a pH of 7.4 was 96.12%, and the simulated wound fluid with a pH of 6.8 was observed to be at 94.73% at 24 h, which corresponds to the sustained delivery of meropenem. Antibacterial studies on *P. aeruginosa*, *S. aureus*, and *E. coli* with meropenem-laden hydrogel showed higher zones of inhibition. The in vivo studies in Sprague Dawley (SD) rats presented accelerated healing with the drug-loaded injectable hydrogel, while 90% wound closure with the unloaded injectable hydrogel, 70% in the positive control group (SC drug), and 60% in the negative control group was observed (normal saline) after fourteen days. In vivo wound closure analysis confirmed that the developed polymeric hydrogel has synergistic wound-healing potential.

## 1. Introduction

Skin serves as the first line defensive barrier of a body against damage, pathogen invasion, and radiation and protects the human body against exogenous harmful factors [1,2]. When the skin becomes compromised, the healing process starts in a physiological fashion, consisting of four overlying stages: hemostasis, proliferation, inflammation, and remodeling to resolve the injury [3,4]. Wounds are the anatomical disruption of skin continuity [5] and constitute the disorganization of dermis composition, leading to the damaging of skin tissue chronically and acutely [6]. Wounds of chronic origin are a worldwide health issue as they do not follow the normal process of wound healing [7], which prolongs the healing time and increases the infection risk and causes serious complications [8]. Problems associated with chronic wound healing are poor oxygenation, age, diabetes, medications, smoking, infection, stress, alcoholism, obesity, and nutrition [9]. Numerous approaches are available for the treatment of wound infections such as microneedles, dressings, foams, films, membranes, liquid dosage forms, conventional therapy, and traditional and modern wound dressings [10]. Among these approaches, bioactive polymer-based thermoresponsive hydrogels consisting of biopolymers have revealed encouraging wound-healing efficacies [11].

Hydrogels are three-dimensional macromolecular networks that can absorb much more water than their dry form and can undergo swelling and expressively undergo volume expansion [12,13,14]. Thermosensitive hydrogels are the stimuli responsive approach, as they show responsiveness to change in the external environment (i.e., temperature); moreover, they undergo swelling due to changes in temperature [2,12,15,16]. These hydrogels exhibit polymers’ shear-thinning properties and lead to the formation of gel from solution [7,17]. Thermosensitive hydrogels have sol–gel alteration above a certain temperature [18]. 

In the current study, we used an FDA-approved triblock copolymer (Pluronic F127) to form the injectable hydrogel. It is composed of a central hydrophobic chain of polypropylene oxide and two hydrophilic side chains of polyethylene oxide. It is used in regenerative medicine due to its ability to form thermoreversible micelles and gels [19]. Hyaluronic acid (HA) is composed of repeating units of *N*-acetyl-d-glucosamine and d-glucuronic acid of nonsulfated glycosaminoglycan [20,21]. HA has a high water sorption capability; retains water; provides lubrication; has an effect on cellular functions such as adhesion, migration, and proliferation; and has various applications in the treatment of joints, tissue regeneration, cosmetics, and ocular surgery [21,22]. Kappa-carrageenan shows distinctive properties that make it an advantageous candidate for use in tissue engineering [23,24]. Hydrogels composed of *κ*-carrageenan increase cell proliferation and cartilage repair [25]. 

The progression of infection in the wound microenvironment is avoided by the use of broad-spectrum antibiotics such as meropenem, which is a β-lactam antibiotic with a broad spectrum of activity. Due to its reduced oral absorption and short half-life, i.e., 0.75–1 h [26], meropenem requires multiple intravenous injections after short intervals, i.e., every 3 h after reconstitution [27]. To improve stability, bioavailability, [28], and patient compliance, a new drug-delivery system for effective meropenem delivery is required [29]. The objective of this study was to develop and evaluate biopolymer-based thermoreversible injectable hydrogels for wound-healing applications and the controlled drug delivery of meropenem and to validate their self-healing potential to stimulate the in vivo wound-healing process in the excisional skin defect model.

In this project, we loaded meropenem, which has a broad spectrum of antibacterial properties, as the model drug, but the main emphasis of this project was to produce an injectable hydrogel composed of a novel biopolymers composite with tissue regeneration properties, i.e., hyaluronic acid and *κ*-carrageenan. Among all the conventional approaches, the bioactive polymer-based thermoresponsive hydrogel consisting of polysaccharides has revealed encouraging wound-healing efficacies. Therefore, we hypothesized that the biopolymer-based injectable hydrogel could stimulate tissue regeneration as well as promote the wound-closure process by providing abundant nutrients at the wound site and enhancing the wound-healing process by protecting the wound from infections. Thermoresponsive sol–gel transitions are beneficial, as they avoid surgical techniques and the complexity of developing biomaterials for transplantation at the site of action.

## 2. Materials and Methods

### 2.1. Materials

Kappa-carrageenan (Mw: 700 kDa) was purchased from TCI (Tokyo, Japan); hyaluronic acid (Mw: 8000–15,000 Da) was purchased from CarboSynth (Berkshire, UK); and Pluronic F-127 and F188 were obtained from Sigma-Aldrich (St. Louis, MO, USA), and were of BioReagent grade. Meropenem was obtained from Zhejiang Ray-bow pharma (Zhejiang, China). Sodium hydroxide, potassium dihydrogen phosphate, hydrochloric acid, potassium chloride, sodium chloride (NaCl), and disodium hydrogen phosphate were purchased from Sigma-Aldrich (St. Louis, MO, USA).

### 2.2. Methodology

#### Development of Thermoreversible Hydrogel

The thermosensitive hydrogels were prepared by using the solvent casting method, also called the cold method [30]. Different solutions of Pluronic F-127, hyaluronic acid, and *κ*-carrageenan were prepared as shown in Table 1. Firstly, Pluronic F-127 was dissolved by adding weighed polymer to distilled water under constant stirring at 4 °C. The resulting solution was marked as solution A. Hyaluronic acid was dissolved separately in distilled water and marked as solution B. A measured quantity of meropenem (1%) was dissolved in solution B. Kappa-carrageenan, marked as solution C, was mixed and continuously stirred in distilled water at 60 °C. Solutions ‘B’ and ‘C’ were mixed at room temperature with constant stirring. Then, this mixed solution was added to Pluronic F-127 solution dropwise and stirred continuously at 4 °C until a homogeneous solution was obtained. The resultant mixture was transported to a glass vial and placed in a water bath maintained at 25°C. The temperature of the water bath was increased gradually up to 37 °C to monitor changes in the formulation [25,31,32].

### 2.3. In Vitro Characterization of Hydrogel

#### 2.3.1. ^1^H NMR and FTIR

Hydrogels were analyzed using ^1^H NMR (Ascend-400MHz, Bruker, Fällanden, Switzerland) operated at 400 MHz for the structural evaluation of the developed formulations. Samples with a weight of 5 mg/mL were dissolved in deuterium oxide and deuterated chloroform, and tetramethylsilane (TMS) was used as an internal standard. Hydrogels were also analyzed for successful crosslinking using using a Nicolet 6700 FT-IR spectrometer (Thermo Scientific, Waltham, MA, USA). The thermosensitive hydrogel was lyophilized by employing LyoDry Freeze Dryers (Edwards Modulyo EF4K freeze dryer, Akribis Scientific Limited, Cheshire, UK), and the dried powder obtained was mixed with KBr in a ratio of 1:100 and subjected to drying. Spectrum was observed at a wavelength of 4000–400 cm^−1^ using an FTIR spectrometer (Nicolet 6700, Thermofisher Scientific, Waltham, MA, USA).

#### 2.3.2. Thermogravimetric Analysis (TGA–DSC)

Thermogravimetric analysis was performed to estimate the thermal stability of the injectable hydrogels and the polymers consumed for the development of gels. Samples were subjected to heating in an ELTRA thermogravimetric analyzer (TGA PT 1000 Linseis, ELTRA GmbH, Haan, Gernany) at the temperature range of 25 °C–500 °C, with a uniform increment of 20 °C per minute at continuous nitrogen flow (20 mL/min). The characterization was performed in triplicate to obtain the thermograms.

#### 2.3.3. Scanning Electron Microscopy (SEM)

The surface morphology and structure of developed injectable hydrogels were determined by using SEM (Tungsten thermionic emission system, 3.5nm and 30keV, Vegas Tescan, Brno, Czech Republic). Samples were prepared for analysis by sprinkling the lyophilized sample powder on the double-sided adhesive tape fixed on the aluminum stub. These stubs were made up to a thickness of ~300 Å with gold coating provided with argon in a high-vacuum evaporator. The surface of the samples was cross-sectionally observed under a 10 kV accelerator current.

### 2.4. In Vitro Studies of Injectable Hydrogel

#### 2.4.1. Gelation Time and Temperature

The thermosensitive hydrogel was poured into vials and placed in the water bath (PolyScience WBE10A11B, PolyScience, Niles, IL, USA) at a temperature of 25 °C. The temperature of the water bath was increased slowly until it reached 37 °C. The flow of the hydrogel in the vial was checked. The time and temperature at which there was no flow in the solution were recorded as gelation time. Values obtained were the average of three determinations [33].

#### 2.4.2. Sol–Gel Phase Transition (T_sol–gel_)

The tube-tilting technique was used for measuring the phase change temperature of the thermosensitive hydrogels. Hydrogels prepared with various ratios of hyaluronic acid, Pluronic F-127, and κ-carrageenan were transferred into glass vials. All vials were kept in a water bath at 25 °C and the temperature was slowly increased to 37 °C. The thermosensitive behavior of developed formulations was evaluated by increasing the temperature [34,35].

#### 2.4.3. Optical Transmittance and Temperature-Induced Change

Optical-transmittance and temperature-induced changes of the hydrogels were measured at different temperatures by using a spectrophotometer (UV-Visible). Transmittance was measured using cuvettes. The temperature of the water bath was slowly increased from 25 °C to a maximum temperature of 40 °C. Before being measured for transmittance, each sample was placed at each temperature for 5 min [36].

#### 2.4.4. Rheological Measurement

An RM200 rotational rheometer (TA-Instruments, Nishigotanda, Japan) was used for the evaluation of the rheological properties of the thermosensitive hydrogels using spindle 5. Rheological behavior was analyzed at different temperatures, 25–34 °C, to assess the effect of increasing temperature on the viscosity of the hydrogels [33,37].

#### 2.4.5. Equilibrium Swelling Ratio

The swelling behavior of the unloaded injectable hydrogels was analyzed in distilled water at 37 °C. All the formulations were properly weighed and placed in distilled water at 37 °C. At specific time intervals, results were recorded until equilibrium was maintained. The percentage swelling index or equilibrium swelling ratio (ESR) of the hydrogels was calculated using:

(1) $$\%\;\mathrm{swelling}\;\mathrm{ratio}=\frac{\left(\mathrm{Ws}-\mathrm{Wi}\right)}{\left(\mathrm{Wi}\right)}$$

where “Wi” is the initial weight and “Ws” is the final weight for the sample.

#### 2.4.6. In Vitro Drug Loading

Meropenem was loaded in the hydrogels by preparing a solution of 15 mg/mL. The drug loading into the hydrogels was performed by adopting the preformulation loading method reported earlier. Briefly, the weighed amount of the drug was dissolved in polymeric hydrogel solution under continuous stirring for 30 min and the mixture was then set up for gel formation in a water bath at 37 ℃ in glass vials.

#### 2.4.7. In Vitro Drug Release and Release Kinetics 

The dialysis bag method was used to carry out in vitro drug release, and a 10 kDa dialysis bag was used. The drug release experiment was performed using an incubator shaker at the speed of 50 rpm and a dialysis membrane. The drug release was performed by incubating the hydrogels in 10 mL of release medium at 37 °C with continuous agitation. The release medium was composed of simulated wound fluid (10 mM) with pH 6.3 and phosphate buffer saline with pH 7.4. The simulated wound fluid (SWF) was composed of 3.3604 g sodium hydrogen carbonate, 5.8440 g sodium chloride, 0.2982 g potassium chloride, 33.00 g bovine albumin, 0.2775 g calcium chloride, and 1000 mL deionized water. Samples with a volume of 1 mL were collected at different time intervals (i.e., 0.5, 1, 2, 4, 6, 8, 10, 12, 16, 20, and 24 h) from the dissolution medium and replaced with 1 mL of fresh medium. The samples collected were then analyzed by a UV spectrophotometer (T80, PG Instruments Limited, Lutterworth, UK) at λ_max_ 298 nm [30]. The first-order, zero-order, Korsmeyer–Peppas, and Higuchi release kinetics models were applied to the drug release data of the injectable hydrogels.

#### 2.4.8. Antibacterial Activity

Antibacterial activity of the thermosensitive hydrogels was evaluated against both Gram-positive (*Staphylococcus aureus)* and Gram-negative bacteria (*Pseudomonas aeruginosa* and *Escherichia coli)* using agar well technique. Agar plates were inoculated with the test microorganisms and four bores were created with the help of a borer. All four bores were separately labeled as drug-loaded injectable hydrogel, drug-unloaded injectable hydrogel, and positive and negative controls, respectively. After the test samples were placed in their respective bores, the plates were incubated for 18–24 h at 37 °C. After 24 h incubation, the zone of inhibition (ZOI) was measured using the following formula:

(2) $$\mathrm{percentage}\;\mathrm{inhibition}=\frac{\mathrm{zone}\;\mathrm{of}\;\mathrm{inhibition}\;\mathrm{of}\;\mathrm{test}\;\mathrm{sample}\;\left(\mathrm{mm}\right)}{\mathrm{zone}\;\mathrm{of}\;\mathrm{inhibition}\;\mathrm{of}\;\mathrm{standard}\;\mathrm{drug}\;\left(\mathrm{mm}\right)}\times 100$$

### 2.5. In Vivo Wound-Healing Analysis

In vivo studies were conducted by using 24 Sprague Dawley rats, weighing 200–250 g and divided into four groups of six rats each. All four groups were individually labeled as the treatment group, the blank hydrogel group, and the positive and negative control groups, respectively. The treatment group was treated with drug-loaded thermoresponsive hydrogels, the blank group was treated with unloaded thermosensitive hydrogel, the negative control group was treated with normal saline, and the positive control group was treated with a subcutaneous injection of meropenem. All groups were provided with standard food, and their temperature conditions were maintained as per the Organization for Economic Co-operation and Development (OECD) guidelines. Rats were anesthetized by administering an intraperitoneal injection of xylazine (15 mg/kg) and ketamine (85 mg/kg). Dorsal hairs were shaved thoroughly, and a 1 × 1 cm excisional wound was created using a surgical blade and forceps.

### 2.6. Wound-Contraction Analysis and Histological Evaluation

The wound closure area was measured and images of the wound site were obtained on day 1, 7, and 14, whereas the percentage wound-closure rate was determined by using the following formula:

wound closure rate % = (A_o_ − A_t_)/A_o_ × 100 (3)

where A_o_ is the initial area of the wound and A_t_ is an area of the wound at a designated time interval. All rats were sacrificed, and a histological evaluation of the wound was carried out by collecting tissue samples on the 1st, 7th, and 14th day after surgery. All samples were collected from the center of the wound and preserved in 10% formalin solution; later on, samples were fixed in paraffin wax. Samples were stained using eosin and hematoxylin dye, and histological images were obtained using a photomicroscope.

## 3. Results

### 3.1. In Vitro Characterization of Hydrogel

#### 3.1.1. ^1^H NMR

**Figure 1:**
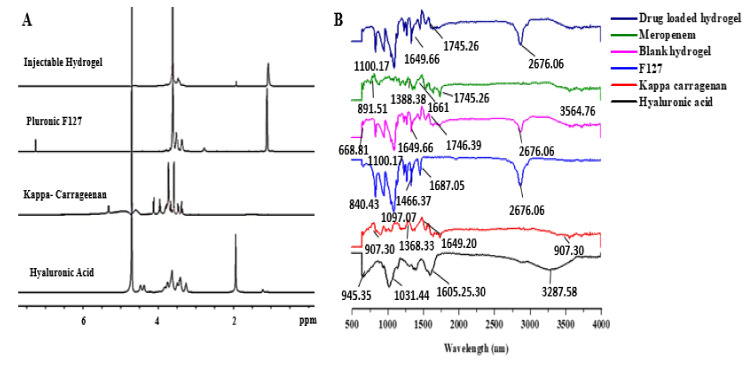
(**A**) ^1^H NMR spectra of the injectable hydrogel, κ-carrageenan, F127, and hyaluronic acid; (**B**) FTIR spectrum of the drug-loaded hydrogel, meropenem, blank hydrogel, F127, κ-carrageenan, and hyaluronic acid.

To investigate and confirm the successful formation of the carrageenan-based hyaluronic acid hydrogel, ^1^H NMR and FTIR spectroscopy was performed. The ^1^H NMR spectrum of F127 showed a distinctive CH_3_ signal of PPO at *δ*_H_ 1.09 ppm (Figure 1A).

#### 3.1.2. FTIR

The FTIR spectra (Figure 1B) of the κ-carrageenan showed that the bands appeared at 847.35, 907.85, 1189.78, and 1208.65 cm^−1^ due to D-galactose-4-sulfate, glycosidic linkage, 3,6-anhydrous-D-galactose, and the ester sulfate stretching of the backbone of κ-carrageenan, respectively [33,34]. The bands at 1541.35, 1564.70, and 1649.20 cm^−1^ were due to functional groups such as carboxamide or carboxylate [35]. These bands are recognized by C=O stretching in carboxamide functional groups and the asymmetric as well as symmetric stretching of carboxylate functional groups. Evidence of the stretching of -OH groups was the broad peak at 3250 cm^−1^ [36]. The FTIR spectroscopy of HA gave several broad bands. At 1031.44 cm^−1^, a band was observed which indicated the presence of C-O-C stretching [37,38]. The presence of the C-O group with C=O was indicated by the band at 1404.52 cm^−1^. Stretching vibrations were observed at 3250 cm^−1^, showing the presence of the OH group [39]. The FTIR of F127 showed bands at 959.38 cm^−1^, indicating the existence of an alkene group (=C-H). The presence of the ether group of the polymer was indicated by the band at 1097.07 cm^−1^ [40,41]. Bands at 1341.34 cm^−1^ were associated with the presence of CH_2_ and CH_3_ groups. 

#### 3.1.3. Thermogravimetric and Differential Scanning Calorimetry Thermographs

**Figure 2:**
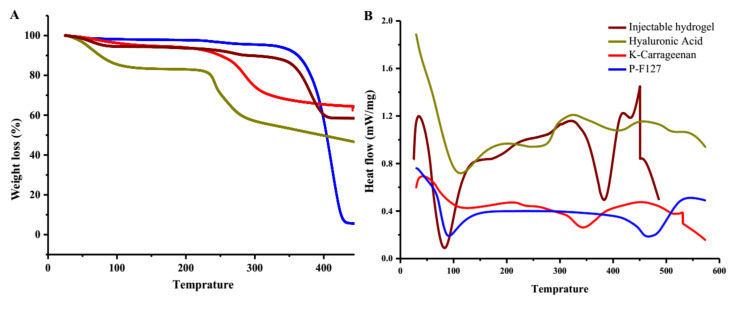
(**A**) Thermogram of F127, hyaluronic acid, κ-carrageenan, and injectable hydrogel; (**B**) differential scanning calorimetry of the injectable hydrogel, hyaluronic acid, κ-carrageenan, and F127.

The thermal stability and degradation of the Pluronic F127, HA, κ-carrageenan, and the injectable hydrogel were estimated using thermogravimetric analysis by a TA analyzer. Figure 2A,B represent the thermographs of the individual polymers and injectable hydrogel. The thermogram of the HA showed 15% weight loss at the temperature range of 50–100 °C, which was associated with the removal of bounded water in the network [42], while the decomposition of the polysaccharide backbone occurred at the temperature range of 250–300 °C, which resulted in 35% weight loss [43]. Similarly, the results from the diffractogram of the HA showed a broad exothermic peak at 100 °C, corresponding to the dehydration of the HA. Another endothermic peak beginning at 150 °C and extending to 300 °C was due to the thermal degradation of the network structure.

The thermogram of the Pluronic F127 represented in Figure 2A demonstrated a weight loss of 10% at 250 °C that was attributed to the elimination of the bound water. The thermal degradation of the triblock polymer accounted for 80% weight loss at the temperature range of 390–450 °C [44,45]. The diffractogram of the Pluronic F127 unveiled an endothermic band at 50 °C to 100 °C that was accredited to moisture loss, as illustrated in Figure 2B, and an endothermic band at 400–500 °C, corresponding to the crystalline chain breakdown [46,47]. 

#### 3.1.4. Scanning Electron Microscopy (SEM)

**Figure 3:**
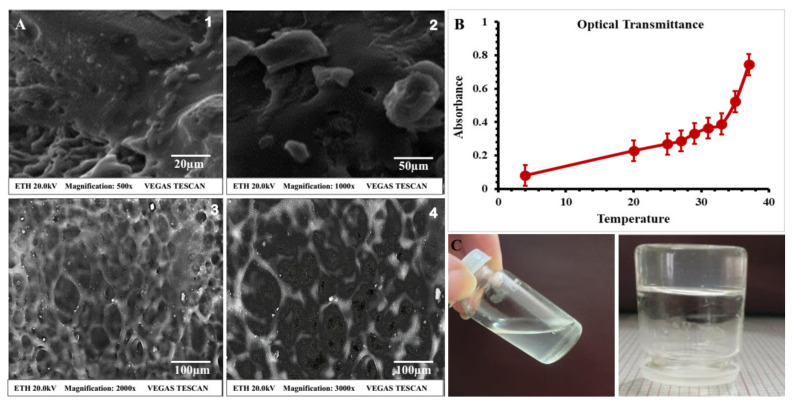
(**A**) Cross-sectional SEM micrograph views of injectable hydrogel at various magnifications to access the porosity in the network: (**1**) at a magnification of 500×; (**2**) 1000× magnification; (**3**) 2000× magnification; and (**4**) magnification 3000×. (**B**) Optical transmittance and temperature-induced changes of the thermosensitive hydrogel at various temperatures. (**C**) Visual representation of transparency of hydrogel and sol–gel phase transition in sol phase of prepared hydrogel and gel phase of prepared hydrogel.

SEM explains the surface morphology and diffusivity details of the synthesized samples. The surface morphology of the hydrogel is presented in Figure 3A. The SEM micrographs show that the synthesized hydrogel surface was porous, dense, and possessed a compact netlike structure. The physically cross-linked injectable hydrogel was examined under different magnifications, and tiny pores in the hydrogel were observed. 

### 3.2. In Vitro Studies

#### 3.2.1. Physical Appearance and Clarity of Thermosensitive Hydrogel

The clarity and physical appearance of the hyaluronic-acid–κ-carrageenan-based thermosensitive hydrogels are shown in Figure 3C.

#### 3.2.2. Gelation Time and Temperature

The goal of this study was to illustrate the gelation time and temperature of the hyaluronic-acid–κ-carrageenan-based F127 injectable hydrogels shown in Table 1. To achieve this goal, different concentrations of polymers were dissolved in distilled water and their gelation time and temperatures were recorded.

#### 3.2.3. Sol–Gel Phase Transition Analysis (T_sol–gel_)

The fabricated hyaluronic-acid–κ-carrageenan-based injectable hydrogels possessed thermoreversible properties. For injectable administration, the hydrogel system should be a liquid at normal room temperature for drug encapsulation and a gel at body temperature. The results in Figure 3C showed that the developed thermosensitive hydrogel could undergo a temperature-dependent change in mechanical strength, and the sol–gel transition was observed upon a change in temperature.

#### 3.2.4. Rheological Study

**Figure 4:**
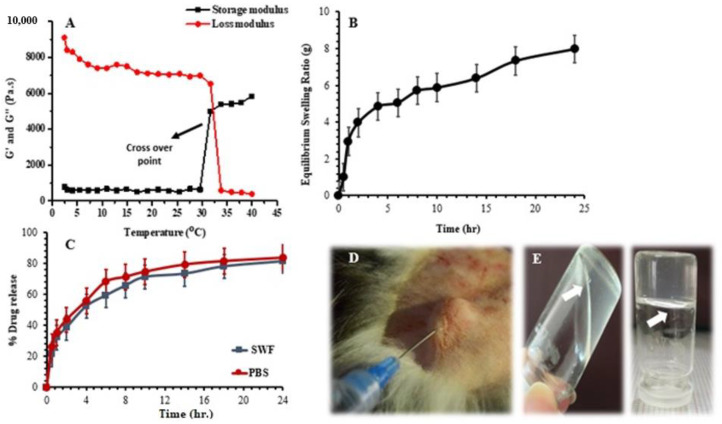
(**A**) Effect of temperature on changing the viscosity of injectable hydrogel by changing the shear rate; (**B**) equilibrium swelling ratio of thermosensitive injectable hydrogel; (**C**) percent drug release vs. time profile of injectable hydrogel; (**D**) in situ gelation after subcutaneous administration of injectable hydrogel in rabbits through 26-guage needle syringe; (**E**) pictorial representation of increasing viscosity due to which the gel faces difficulty in flowability.

The thermosensitive injectable hydrogel was measured for its rheological properties to check the flow of the formulated hydrogel, and the results are shown in Tables S1 and S2 (Supplementary Data). The rheology of the hydrogel was assessed at two different temperatures with an increasing shear rate, i.e., 25 °C and 34 °C. The results indicated that the solution viscosity increased with the increase in temperature, i.e., the viscosity and temperature were directly proportional to each other, as shown in Figure 4A.

#### 3.2.5. Optical Transmittance and Temperature-Induced Change

The optical transmittance and temperature-induced changes of the hydrogels were measured at different temperatures by using a UV-vis spectrophotometer (UV-1280, Shimadzu, Kyoto, Japan), as shown in Figure 3B. 

#### 3.2.6. Equilibrium Swelling Ratio (ESR)

The swelling behavior of the synthesized injectable hydrogels was analyzed in distilled water at 37 °C [48]. When water molecules come in contact with such hydrophilic groups, the network structure becomes hydrated due to the increased uptake of water molecules. The formulations encoded as HC-1, HC-2, and HC-3 with increasing hyaluronic acid concentrations, i.e., 3%, 4%, and 5% *w/v*, showed increased swelling. Swelling is greater in distilled water because of the ionization of the carboxyl group; consequently, the counterion concentration is increased within the network.

#### 3.2.7. In Vitro Drug-Release Studies

Drug-release studies were conducted in simulated wound fluid (SWF) and phosphate buffer saline (PBS), as shown in Figure 4C. The percentage drug release in the SWF was calculated and was found to range between 81.542 and 94.736%. The minimum drug release was observed for the formulation HC-9 (81.542%), and the maximum release was observed for the HC-3 formulation (94.736%). The drug release in PBS was slightly higher as compared to the SWF, i.e., it ranged from 83.76 to 96.12%. The maximum drug release was observed in HC-7 and HC-9 (83.76%). This indicated that the amount of meropenem released in PBS was slightly higher than the amount released in SWF.

#### 3.2.8. Drug-Release Kinetics

Drug release from hydrogels is a complex process; therefore, to estimate the drug-release kinetics, different kinetic models were used. The zero-order, first-order, Higuchi, and Korsmeyer–Pappas model results are shown in Table S3 (Supplementary Data). The drug-release mechanism of the hydrogel was explained by values close to the regression line, and it did not involve the mechanism of swelling.

#### 3.2.9. Antibacterial Activity

**Figure 5:**
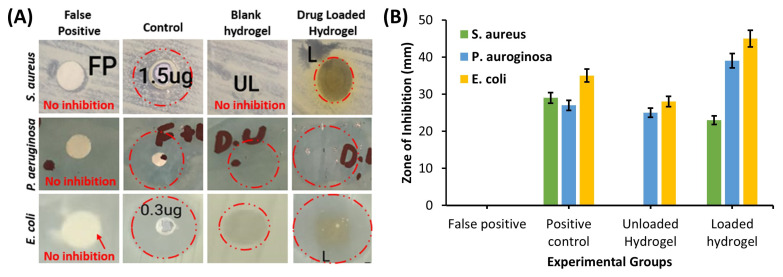
(**A**) Zone of inhibition against *S. aureus*, *P. aregnosa* and *E. coli* in positive control, negative control, blank hydrogel, and drug-loaded hydrogel; (**B**) graphical representation of zone of inhibition observed.

The antibacterial activity of the thermosensitive hydrogels was measured by the agar well method [49]. A clear zone of inhibition (ZOI) was observed against *S. aureus*, *P. aregnosa,* and *E. coli* for the negative control (normal saline), positive control (meropenem), blank hydrogel, and drug-loaded hydrogel, as shown in Figure 5A,B.

### 3.3. In Vivo Wound-Healing Analysis

#### 3.3.1. Animal Studies

**Figure 6:**
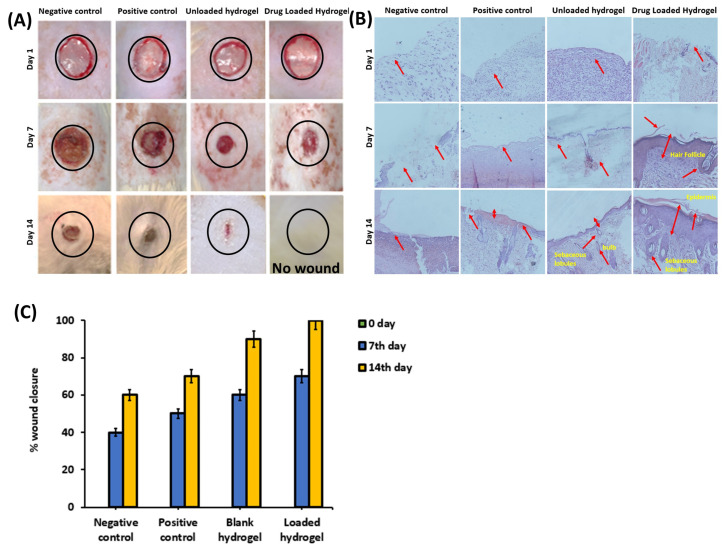
(**A**) Macroscopic appearance of wounds post-surgery in four groups at 0th 7th and 14th day of the incision. (**B**) Wound section histology of different groups on 0, 7th and 14th days stained with H & E stain. (**C**) Wound closure of different groups on 0, 7th, and 14th day of the incision.

The percentage wound closure was determined by measuring the area of the wound, as shown in Figure 6A. The percentage of wound closure of the blank injectable hydrogel, as well as the drug-loaded injectable hydrogel, was greater than that of the positive and negative control groups.

#### 3.3.2. Histological Examination

Histological studies of the negative and positive controls, the blank hydrogel groups, and the drug-loaded hydrogel group were carried out at zero, seven, and fourteen days for wound-healing analysis, and the results are shown in Figure 6B.

## 4. Discussion

### 4.1. In Vitro Characterization of Hydrogel

#### 4.1.1. ^1^H NMR

The peak at *δ*_H_ 3.60 ppm showed the presence of a hydroxymethyl proton [50,51,52]. A singlet was observed at *δ*_H_ 1.94 ppm in the ^1^H NMR spectrum of the HA that indicated the presence of *N*-acetyl protons (NCO*C*H_3_). The multiplets observed at *δ*_H_ 3.20–3.90 ppm showed the presence of protons from sugar moiety [53], and the κ-carrageenan spectrum revealed a peak at *δ*_H_ 3.56 ppm indicating O-methylene protons because of the presence of 3-linked 6-O-methyl-D-galactose residue present in κ-carrageenan [54]. Mahmood et al. observed a similar effect with slight variation in chemical shift, which might be due to the difference in the source of the polymer obtained [55]. Compared with the spectra of the polymers, the injectable hydrogel showed a peak at *δ*_H_ 1.07 ppm, which was also present in the spectrum of F127, belonging to the methyl group of PPO in F127. The signal at *δ*_H_ 3.56 ppm belonged to the O-methylene group of κ-carrageenan, while the ^1^H NMR experiment was carried out as underwater suppression pulse program and the signals at *δ*_H_ 1.94 ppm for the HA were present in a small concentration. These observations suggested that no chemical interaction occurred between the polymers, and that the Pluronic F-127 was a major part of the self-assembled hydrogel which helped in the gel formation, while all the other components of the hydrogel were incorporated as minor part to enhance the tissue repair and regeneration. 

#### 4.1.2. FTIR

The alkyl group presence was indicated by the formation of the band at 841.08 cm^−1^ in the formulation. In the case of the injectable hydrogel, bands occurred at 1240.17, 1341.62, 1466.16, and 1540.88 cm^−1^. Some bands appeared at 1208.65, 541.35, and 1564.70 cm^−1^ in the κ-carrageenan. The FTIR of the meropenem showed peaks at 1007.13, 1092.76, 1187.77, 1256.89, and 1388.38 cm^−1^, which corresponds to the presence of carbonyl CO stretching [56,57]. The band at 2877.10 showed the presence of C-H stretching, the band at 1617.80 was due to –NH bending vibrations, and the peak at 1745.26 showed the presence of the carboxylic group in the structure. In the meropenem-loaded carrageenan-based hyaluronic acid hydrogel, the existence of meropenem was confirmed by the presence of its characteristic peaks responsible for the pharmacological effect.

#### 4.1.3. Thermogravimetric and Differential Scanning Calorimetry Thermographs

The TGA of the κ-carrageenan revealed the two-stage degradation phenomena, as demonstrated in Figure 2A. The onset of the first stage began at room temperature and extended up to 100 °C, and was related to the loss of moisture from the hydrated polymer, as bioactive polymers have a strong affinity for water [58], whereas the total loss for the second degradation phase at the temperature range of 250–325 °C was 25%, which corresponds to the degradation of the polysaccharide skeleton. The diffractogram of the κ-carrageenan is shown in Figure 2B, which represented the loss of free and bound water at 25–75 °C and 100 °C, respectively, while the exothermic peak at 310 °C was considered as the Tg, suggesting the decomposition of the D-galactose ring [59]. 

Additionally, Figure 2A,B show the thermograms and Tg of the freeze-dried injectable hydrogel. The gravimetric analysis revealed that the initial weight loss was low (i.e., 5%) at 100 °C, which was associated with the loss of bounded water, while only 40% of the total weight loss occurred at the degradation temperature (Tg) 350 °C, indicating that the formulation had a higher thermal stability compared to the individual components HA, κ-carrageenan, and Pluronic F127. Furthermore, the injectable hydrogel presented an exothermic peak at 50–75 °C, attributed to moisture loss, immediately followed by an endothermic peak extending from 75 °C to 400 °C, which was attributed to the existence of a unique Tg curve that may have corresponded to successful self-assembly. This shift of the glass transition values of the injectable hydrogel compared to the polysaccharide polymers was related to physical crosslinking in the polymers.

#### 4.1.4. Scanning Electron Microscopy (SEM)

Small pores appeared in the structure due to the F127, which supported the diffusion of water molecules around the structure. This also showed the greater influence of the polymers in the network structure and explained the greater interaction of the polymers within the hydrogel [60]. The expanded structure achieved a more stable and diffusible hydrogel, and the porous network could hold more water, facilitating the swelling of the hydrogel; similarly, the tiny pores on the surface suggested that the drug release from the hydrogel would occur in a sustainable manner [61].

### 4.2. In Vitro Studies

#### 4.2.1. Physical Appearance and Clarity of Thermosensitive Hydrogel

The clarity and physical appearance of the HA–Cr-based F127 polymeric blend and the thermosensitive hydrogel were observed visually. The developed hydrogels had a transparent appearance and soft texture. A higher concentration of κ-carrageenan resulted in a slightly cloudy solution, while a greater concentration of hyaluronic acid enhanced the transparency of the developed hydrogels.

#### 4.2.2. Gelation Time and Temperature

The gelation time of three hydrogels, i.e., HC-1, HC-2, and HC-3, with different concentrations of hyaluronic acid (3%, 4%, and 5% *w/v*) were noted. It was observed that by increasing the concentration of hyaluronic acid, the required time for gelation decreased. HC-1 took 13 s to become a gel, whereas HC-2’s gelation time was 10 s and HC-3’s gelation time was 8 s, as shown in Table 1. The gelation temperatures of HC-1, HC-2, and HC-3 were 36 °C, 35 °C, and 34 °C, respectively. The decrease in the gelation time indicated a microstructural change in the formulation due to the interaction between the hyaluronic acid and the F127 [62]. In an aqueous solution above critical micelle concentration, the self-assembly of copolymer molecules into micelles takes place. By increasing the temperature, the micelles pack together and overlap each other by hydrophobic interactions of the PPO blocks [63]. Low-molecular-weight hyaluronic acid, as was used in this study, allows the packing and movement of micelles, causing a change in the gelling properties [64]. 

The concentration of *κ*-carrageenan in the HC-4, HC-5, and HC-6 hydrogel formulations increased by 0.1%, 0.2%, and 0.3% *w/v*, respectively. It was observed that by increasing the concentration of *κ*-carrageenan, the required time for gelation increased. The gelation times of HC-4, HC-5, and HC-6 were recorded 9 s, 10 s, and 12 s, respectively, while the gelation temperatures of HC-4, HC-5, and HC-6 were noted as 33 °C, 35 °C, and 36 °C, respectively, as shown in Table 1. By increasing the concentration and temperature, the coil-to-helix structure of *κ*-carrageenan begins to shift towards a helix-to-coil conformation. Due to this transition, the melting of the helices and the structural rearrangement results in an increased gelation time and temperature [65].

The concentration of Pluronic F127 in the HC-7, HC-8, and HC-9 hydrogel formulations increased by 21%, 23%, and 25% *w/v*, respectively. Pluronic F127 in a concentration above 15% undergoes thermoreversible gelation, which is crucial for injectable administration [66]. It was observed that by increasing the concentration of F127, the gelation time and temperature decreased. The mechanism of the gelation of Pluronic F127 depends on the packing and entanglement of micelles. With the increase in temperature, micelles come into close contact and do not move. The micelles do not separate easily from each other due to micelle entanglements, and they form a rigid gel [67]. The critical micelle concentration and temperature were changed by the addition of hyaluronic acid and *κ*-carrageenan; hence, the gelation temperature of the developed hyaluronic-acid–κ-carrageenan-based F127 hydrogel system should be higher than pure Pluronic F127. In the meropenem-loaded hydrogel, the concentration of meropenem was constant in all the formulations, from HC-1 to HC-9, i.e., 1%. A slight increase in the gelation time and temperature was observed after drug loading into the hydrogel. Meropenem is a hydrophilic drug, and when it was loaded into the F127-based thermosensitive hydrogel, it resulted in the increased hydrophilicity of the system, causing a slight change in its gelation properties [67].

#### 4.2.3. Sol–Gel Phase Transition Analysis (T_sol–gel_)

The injectable hydrogel with a Pluronic F127 concentration lower than 15% did not undergo gelation at body temperature. Pluronic F127 is composed of polypropylene oxide and polyethylene oxide (PEO–PPO–PEO). PEO side chains are hydrophilic, while the PPO in the center is hydrophobic [68]. The hydrophilic PPO core becomes exposed, resulting in gelation and increased chain entanglements. When the concentration of F127 is increased, these chain entanglements also increase, resulting in the hydrogel. The developed hydrogel responded to temperature change, achieving the flowing phase at 25 °C and nonflow state at 37 °C, thus conforming to sol–gel phase transition. Sol–gel transition is an ideal parameter for injectable hydrogel, while instant gelation leads to the clogging of the syringe before administration 

#### 4.2.4. Rheological Study

The hydrophilic interactions were overcome by hydrophobic interactions when the temperature was increased above LCST, resulting in a dehydrated state with an increase in viscosity [69,70]. The storage moduli and loss moduli of the hyaluronic-acid–κ-carrageenan (HA–κC)-based injectable hydrogel are presented in Figure 4A. The grafting process of the injectable hydrogels was assumed to be over when the G′ extended to a plateau formation. The grafting of the κ-carrageenan and HA to the F127 in the HA–κC hydrogel was completed when the G′ extended the G″, where the cross-over point represents the gelation. The crossover point was situated at 32 °C, which corresponded to an upsurge in the viscosity with the function of changing temperature. Moreover, the increasing temperature led to a reduction in the time it took to reach the plateau value and an increase in the storage modulus [71]. Additionally, the damping factor of the samples was between 0.001 and 0.003. The damping factor for the HA–κC hydrogel was low, attributable to the viscoelastic behavior of injectable hydrogels [72,73].

#### 4.2.5. Optical Transmittance and Temperature-Induced Change

The transmittance of the fabricated hydrogel was measured by using cuvettes. The developed thermosensitive hydrogel demonstrated an increase in absorbance beyond the critical gelation temperature (≥32 °C). The increase in absorbance was due to the larger particle size at a temperature higher than LCST. It was observed that below LCST, the hydrogel solution was transparent due to the hydrophilic interactions, which indicated the uniform solubility of the added components. After increasing the temperature, the hydrophilic interactions were overcome by hydrophobic interactions, resulting in the increased size of the components and the turbidity of the solution [74].

#### 4.2.6. Equilibrium Swelling Ratio (ESR)

Upon ionization, the osmotic pressure difference arose within the solution and outside the gel, resulting in greater swelling [75,76]. When the concentration of κ-carrageenan was increased, i.e., 0.1% in HC-4, 0.2% in HC-5, and 0.3% in HC-6, it was observed the swelling of the hydrogel also increased, as shown in Figure 4B. The sulfate groups in the κ-carrageenan structure are ionizable, and they become deprotonated to produce OSO_3_^−^ in the system. Therefore, as the concentration of the polymer increased, the number of these negatively charged groups also increased, resulting in greater repulsion and increased swelling. Negatively charged groups on different chains induce electrostatic repulsion, due to which the distance between the chains also increases. As a result, the space between the network becomes larger and more permeable to larger molecules. Hence, a large amount of water can penetrate the polymeric network, which results in greater swelling [76]. On the other hand, upon increasing the concentration of F127, a decrease in swelling was observed, as shown in Figure 4B, possibly because the PEO side chains are hydrophilic while the PPO in the center is hydrophobic in the structure of Pluronic F127. When hydrogen bonds break, the hydrophilic side chains become weak [77]; as a result, when the concentration of F127 was increased, these chain entanglements also increased, resulting in greater gel strength. Less water could penetrate inside the gel, which ultimately resulted in less swelling. Upon increasing the concentration of F127, the hydrophobic groups in the center involved in intermolecular entanglements increased, creating a transient 3D polymer network. This phenomenon provided strength to the gel, decreasing swelling by hindering the passage of water molecules [78].

#### 4.2.7. In Vitro Drug-Release Studies

In the formulations where the hyaluronic acid concentration was increased from 3% *w/v* to 5% *w/v* in PBS and SWF, an increase in the concentration of HA to 5% resulted in better drug release. The ionization of the carboxyl group influences the counterion concentration, and with an increased HA concentration more counterions are generated, resulting in the creation of an osmotic pressure difference between the solution within and outside the gel, which is responsible for better drug release [79]. Increasing the concentrations of κ-carrageenan from 0.1% *w/v* in HC-4 to 0.2% *w/v* in HC-5 and 0.3% *w/v* in HC-6 changed the charge density on the hydrogel network due to the presence of ionic groups, i.e., COO^−^ and OSO_3_^−^. As the concentration of carrageenan increased, these groups are repelled by each other as well as solvent molecules, resulting in the swelling of the hydrogel and increased drug release [80]. The deprotonation of the OSO_3_H and COOH groups also occurs, resulting in the maximum ionization of these groups, which causes increased swelling and drug release from the matrix [81]. Drug-release studies were performed on the hydrogel with increasing concentrations of F127 from 21% *w/v* in HC-7 to 23% *w/v* in HC-8 and 25% *w/v* in HC-9. It was observed that in both dissolution media, the drug release decreased with an increasing F127 concentration; this was because of the influence of the hydrophobic groups that are present in the F127 structure [82]. When its concentration increased, micelle formation also increased, due to the extensive interaction between the hydrophobic groups which in turn thickened the gel layer and hindered the drug release from the gel matrix [83].

#### 4.2.8. Drug-Release Kinetics

The Higuchi model shows a high R^2^ value, so it better fit the regression line and more effectively explained the drug-release mechanism from the matrix [84]. The Higuchi model is based on two hypotheses: firstly, the drug concentration present in the matrix is greater than the drug solubility; secondly, the flow of the drug in the matrix system is one-dimensional. According to this model, the drug particle size is lower than the system thickness, and there is constant diffusion of the drug during its release [84]. The Korsmeyer–Peppas model also explained the drug release from the polymer. The value of “n” was calculated by plotting the values of the release data in the Korsmeyer–Peppas model sheet. If the value of n was equal to or less than 0.45 (n ≤ 0.45), then the Fickian diffusion model was followed.

#### 4.2.9. Antibacterial Activity

Figure 5A depicts that the ZOI observed against *S. aureus* was 28.4 mm for the positive control, whereas no zone was observed for the negative control. Moreover, the ZOI obtained for the blank injectable hydrogel was 3.7 mm, whereas a zone of 30 mm was observed for the drug-loaded injectable hydrogel. For *P. areginosa*, no inhibition zone was observed in the negative control group, whereas ZOIs of 18.66 mm, 6.33 mm, and 20.66 mm were observed in the positive control, blank hydrogel, and drug-loaded hydrogel, respectively. In the case of *E. coli,* the ZOIs observed for the blank and drug-loaded hydrogel were 27.96 mm and 22.6 mm, respectively. On the other hand, no ZOI was observed for the blank hydrogel and negative control groups. The study results demonstrated that the blank injectable hydrogels had little or no antibacterial activity, while clear ZOIs were observed against all the bacterial strains in the drug-loaded injectable hydrogel, as shown in Figure 5A. It was observed from the study results that the drug-loaded membrane showed a larger ZOI against the Gram-positive strain compared to the Gram-negative strain, which might have been due to the cell-wall structure of Gram-negative bacteria. Gram-negative bacteria contain an outermost membrane of peptidoglycan, which protects them against environmental damage.

### 4.3. In Vivo Wound-Healing Analysis

#### 4.3.1. Animal Studies

During the first week of the wound-healing analysis, little inflammation in all the rats was observed [85]. No signs of infection were observed, and crusts started to form by the end of the first week, which indicated the epithelization process in all groups. On day 14, both the positive and negative control groups showed slower epithelialization, with 70% and 60% wound closure, respectively. Contrastingly, a marked reduction in wound size was observed in the blank and drug-loaded hydrogel groups, with 90% and 100% wound closure, respectively. The fast healing potential displayed in the blank and drug-loaded hydrogel groups was attributed to the presence of HA and *κ*–carrageenan, which has an effect on the different stages of wound healing, such as migration, adhesion, and proliferation [21,22]. Moreover, the sustained release of meropenem prevented the wound from secondary infection and aided in faster healing.

#### 4.3.2. Histological Examination

At zero days, very few inflammatory cells were observed, which was referred to as the chronic inflammatory phase in all four groups. On the seventh day of the experiment, small blood vessels were emerging in the wound microenvironment in the drug-loaded hydrogel group and a negligible amount of inflammatory cells were seen compared to the negative and positive control groups, whereas the unloaded injectable hydrogel group also showed few inflammatory cells, showing that the inflammatory phase was complete after seven days in the drug-loaded injectable group [86,87]. Histological analysis confirmed the presence of abundant fibroblasts and granulating cells in the injectable hydrogel groups, while few were present in the control groups [88].

On the fourteenth day, there were still several neovascularization and inflammatory cells present in the other two groups, particularly the negative control group. The drug-loaded injectable hydrogel group showed a thicker granulating layer than the unloaded injectable group, while the negative control showed a thinner granulating layer than the positive control group. Angiogenesis was observed in the drug-loaded and drug-unloaded injectable hydrogel groups, while it was still absent in the control groups. The histological analysis results showed abundant mature collagen in the drug-loaded and unloaded hydrogel groups, whereas immature collagen was found in the positive and negative groups. On the 14th day, sebaceous glands, sweat glands, and hair follicles were observed, as shown in Figure 6B, whereas no such glands were observed in the positive and negative control groups. The histopathology results showed that in the control groups, the healing process was slower and delayed, while the hydrogel groups showed faster healing by employing re-epithelialization and ECM deposition and remodeling.

## 5. Conclusions

In this research, an in situ injectable biopolymer-based hydrogel network was developed to stimulate the wound-healing cascade in an excisional wound model. For improved drug delivery and wound healing, the cold method was employed, using Pluronic F127 and bioactive polymers with commendable biocompatibility to form in situ injectable hydrogels with prompt gelation and tunable mechanical properties. The structure of the cross-linked injectable hydrogel was confirmed through FTIR. The thermal stability was evaluated through TGA and DSC, while the hydrogel’s porous structure was analyzed using SEM. Swelling studies showed that the system swelled accurately, while release studies demonstrated the efficient release of the meropenem from the thermosensitive hydrogel matrix. All the results proved that the proposed hydrogel solution could be useful for wound healing and that this developed hydrogel has the potential to act as a bioactive wound healer for the synergistic improvement of impaired skin wound healing.

## Figures and Tables

**Table 1:** Hyaluronic-acid–κ-carrageenan-based F127 thermosensitive hydrogel feed composition.

Formulation	HA (%*w*/*w*)	κ-C (%*w*/*w*)	F127 (%*w*/*w*)	Meropenem	Gel Temp	Gel Time
				(% *w*/*w*)	(°C)	(s)
HC-1	3	0.2	21	1	36	13
HC-2	4	0.2	21	1	35	10
HC-3	5	0.2	21	1	34	8
HC-4	4	0.1	21	1	33	9
HC-5	4	0.2	21	1	35	10
HC-6	4	0.3	21	1	37	12
HC-7	4	0.2	21	1	36	15
HC-8	4	0.2	19	1	33	10
HC-9	4	0.2	21	1	31	6

## Data Availability

Not applicable.

## Supplementary Materials

The following supporting information can be downloaded at: https://www.mdpi.com/article/10.3390/polym14030376/s1, Table S1: Rheological study results for thermosensitive injectable hydrogel at 25 °C, Table S2: Rheological study results for thermosensitive injectable hydrogel at 34 °C, Table S3: Release kinetic models of injectable hydrogel with changing HA, kappa-carrageenan, and F127 concentration.
