# Correction: Ijaz et al. Biofunctional Hyaluronic Acid/κ-Carrageenan Injectable Hydrogels for Improved Drug Delivery and Wound Healing. *Polymers* 2022, *14*, 376

**DOI:** 10.3390/polym17091278

**Published:** 2025-05-07

**Authors:** Uzma Ijaz, Muhammad Sohail, Muhammad Usman Minhas, Shahzeb Khan, Zahid Hussain, Mohsin Kazi, Syed Ahmed Shah, Arshad Mahmood, Mohammed Maniruzzaman

**Affiliations:** 1 Department of Pharmacy, Abbottabad Campus, COMSATS University Islamabad, Abbottabad 22010, Pakistan; uzmaijaz92@gmail.com (U.I.); syedahmed.shah@superior.edu.pk (S.A.S.); 2 College of Pharmacy, University of Sargodha, Sargodha 40100, Pakistan; usman.minhas@uos.edu.pk; 3 Department of Pharmacy, University of Malakand, Chakdara 18800, Pakistan; shahzebkhan@uom.edu.pk; 4 Discipline of Pharmaceutical Sciences, School of Health Sciences, University of KwaZulu-Natal, Durban 4041, South Africa; 5 Department of Pharmaceutics & Pharmaceutical Technology, College of Pharmacy, University of Sharjah, Sharjah P.O. Box 27272, United Arab Emirates; zhussain@sharjah.ac.ae; 6 Research Institute for Medical and Health Sciences (SIMHR), University of Sharjah, Sharjah P.O. Box 27272, United Arab Emirates; 7 Department of Pharmaceutics, College of Pharmacy, King Saud University, P.O. Box 2457, Riyadh 11451, Saudi Arabia; mkazi@ksu.edu.sa; 8 Department of Pharmaceutical Sciences, The Superior University, Lahore 54600, Pakistan; 9 College of Pharmacy, Al Ain University, Abu Dhabi P.O. Box 112612, United Arab Emirates; arshad.mahmood@aau.ac.ae; 10 Division of Molecular Pharmaceutics and Drug Delivery, Department of Molecular Pharmaceutics and Drug Delivery, College of Pharmacy, The University of Texas at Austin, Austin, TX 78712, USA; m.maniruzzaman@austin.utexas.edu

In the original publication [1], there was a mistake in Figures 5 and 6 as published. Due to the high workload, a research scholar involved in this specific project mistakenly swapped images of antibacterial and histological assays (Figures 5 and 6). The corrected Figure 5 and Figure 6 appear below. The authors state that the scientific conclusions are unaffected. This correction was approved by the Academic Editor. The original publication has also been updated.

The revised figures have been included in the updated manuscript in the attachment.

## Figures and Tables

**Figure 5:**
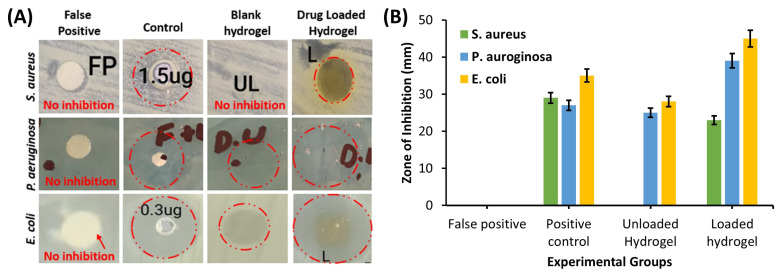
(**A**) Zone of inhibition against *S. aureus*, *P. aregnosa* and *E. coli* in positive control, negative control, blank hydrogel, and drug-loaded hydrogel; (**B**) graphical representation of zone of inhibition observed.

**Figure 6:**
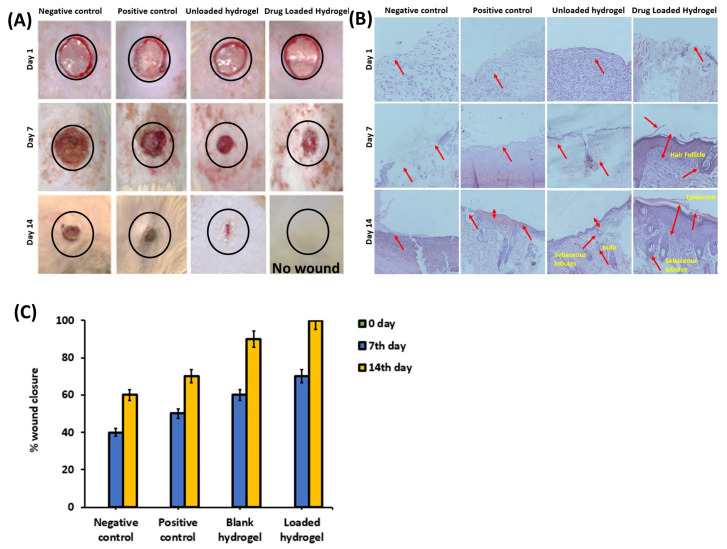
(**A**) Macroscopic appearance of wounds post-surgery in four groups at 0th 7th and 14th day of the incision. (**B**) Wound section histology of different groups on 0, 7th and 14th days stained with H & E stain. (**C**) Wound closure of different groups on 0, 7th, and 14th day of the incision.
