# Supplementary material for: Biofunctional Hyaluronic Acid/κ-Carrageenan Injectable Hydrogels for Improved Drug Delivery and Wound Healing
Source: Polymers (Basel). 2022 Jan 18;14(3):376. doi: 10.3390/polym14030376 (PMC8840380; doi:10.3390/polym14030376)
Supplement: Supplementary file 1 [file polymers-14-00376-s001.zip › polymers-1526549-supplementary.pdf]

Table S1. Rheological study results for thermosensitive injectable hydrogel at 25 ºC.

S. No	Temp (ºC)	Time (sec)	Shear rate D(s-1)	Shear stress (Pa)	Viscosity (Pa.s)
1	25	11.4	10	0.188	0.019
2	25	21.4	20	0.0128	0.0064
3	25	31.4	30	0.144	0.0048
4	25	41.4	40	0.014	0.0035
5	25	51.4	50	0.045	0.0026
6	25	61.4	60	0.05	0.002
7	25	71.4	80	0.161	0.0019
8	25	81	100	0.111	0.0017
9	25	91.4	120	0.13	0.0015
10	25	101.4	140	0.211	0.0014
11	25	111.4	180	0.186	0.0013
12	25	121.4	220	0.256	0.0011
13	25	131.4	260	0.341	0.0013
14	25	141.4	300	0.44	0.001
15	25	151	340	0.551	0.0008

Table S2. Rheological study results for thermosensitive injectable hydrogel at 34 ºC.

S. No	Temp ºC	Time (sec)	Shear rate D(s-1)	Shear stress(Tau(Pa)	Viscosity (Pa.s)
1	25	11.4	10	4.64	0.464
2	25	21.4	20	4.42	0.221
3	25	31.4	30	4.73	0.158
4	25	41.4	40	4.51	0.113
5	25	51.4	50	4.11	0.08
6	25	61.4	60	3.53	0.059
7	25	71.4	80	2.66	0.033
8	25	81	100	2.4	0.024
9	25	91.4	120	2.51	0.021
10	25	101.4	140	2.46	0.018
11	25	111.4	180	2.53	0.014
12	25	121.4	220	2.86	0.013
13	25	131.4	260	3.32	0.013
14	25	141.4	300	3.78	0.013
15	25	151	340	4.5	0.013

Table S3. Release kinetic models of injectable hydrogel with changing HA, kappa carrageenan and F127 concentration.

Sample Codes	Simulated body fluids	Zero Order kinetics	First Orders kinetics	Higuchi Model	Korsmeyer peppas
		R^2	R^2	R^2	n
HC-1	SWF	0.8871	0.9865	0.9792	0.975
	PBS	0.8242	0.9663	0.9482	0.9423
HC-2	SWF	0.9067	0.9949	0.9844	0.9756
	PBS	0.8563	0.9823	0.9637	0.9677
HC-3	SWF	0.882	0.9909	0.9758	0.9629
	PBS	0.8328	0.9927	0.954	0.9569
HC-4	SWF	0.8955	0.9869	0.9828	0.9856
	PBS	0.8671	0.9761	0.977	0.9875
HC-5	SWF	0.8602	0.9616	0.9628	0.9829
	PBS	0.8299	0.959	0.9529	0.9786
HC-6	SWF	0.8982	0.9898	0.9795	0.97719
	PBS	0.8887	0.9906	0.9767	0.981
HC-7	SWF	0.9299	0.9886	0.9811	0.9673
	PBS	0.8869	0.977	0.9668	0.9627
HC-8	SWF	0.8275	0.9716	0.955	0.9821
	PBS	0.7202	0.9168	0.8845	0.9349
HC-9	SWF	0.7868	0.913	0.9322	0.9744
	PBS	0.7433	0.8766	0.9047	0.9712
